# Roles of lncRNAs in influenza virus infection

**DOI:** 10.1080/22221751.2020.1778429

**Published:** 2020-06-18

**Authors:** Jing Wang, Shan Cen

**Affiliations:** a Institute of Medicinal Biotechnology, Chinese Academy of Medical Sciences and Peking Union Medical School, Beijing, People's Repbulic of People’s Republic of China; b CAMS Key Laboratory of Antiviral Drug Research, Peking Union Medical College, Chinese Academy of Medical Sciences, Beijing, People's Repbulic of People’s Republic of China; c Beijing Friendship Hospital, Capital Medical University, Beijing, People's Repbulic of People’s Republic of China

**Keywords:** LncRNAs, influenza virus, host immune response, virus-host interaction, virus infection

## Abstract

Recent studies have identified host long noncoding RNAs (lncRNAs) as key regulators of host-virus interactions during viral infection. The influenza A virus (IAV) remains a serious threat to public health and economic stability. It is well known that thousands of lncRNAs are differentially expressed upon IAV infection, some of which regulate IAV infection by modulating the host innate immune response, affecting cellular metabolism, or directly interacting with viral proteins. Some of these lncRNAs appear to be required for IAV infection, but the molecular mechanisms are not completely elucidated. In this review, we summarize the roles of host lncRNAs in regulating IAV infection and provide an overview of the lncRNA-mediated regulatory network. The goal of this review is to stimulate further research on the function of both well-established and newly discovered lncRNAs in IAV infection.

## Introduction

It is estimated that more than 70% of the human genome is transcribed into RNA, but only 2% encodes proteins [1,2]. These non-coding RNAs (ncRNAs) have been classified, based on their lengths, into small ncRNAs (< 200 nucleotides) and long noncoding RNAs (>200 nucleotides). The latter are referred to as lncRNAs. Most annotated lncRNAs are transcribed by RNA polymerase II (Pol II) and are 5′-capped, spliced and polyadenylated, although alternative 3′-topologies are also occasionally observed [3,4]. LncRNAs can be generated from stand-alone transcriptional units, enhancers, promoters, introns of other genes, antisense strands of other genes, or pseudogenes [5,6]. Many lncRNAs are less evolutionarily conserved, less abundant than mRNA (only about 10% of the median mRNA level), and exhibit more time- or space-specific expression [7,8]. However, recent data show that lncRNAs can regulate the expression of protein-coding genes at the levels of chromatin remodelling and transcriptional and post-transcriptional processing. They play key roles in various biological processes, including cell-cycle regulation, apoptosis, and cell differentiation [9–12]. Recent studies have also identified lncRNAs as important regulators of virus-host interactions [13–17]. This review highlights the involvement of specific lncRNAs in the pathogenesis of influenza virus from the mechanistic point of view.

## Cellular lncRNA expression is altered by IAV infection

To establish a productive infection, influenza viruses manipulate host factors to promote virus replication and to suppress host antiviral responses. The first analysis of the widespread differential expression of lncRNAs in response to IAV infection revealed that most lncRNAs which are differentially expressed during severe acute respiratory syndrome coronavirus (SARS-CoV, MA15) infection are similarly regulated by IAV (A/PR/8/1934) infection in mice [18]. Perturbation of innate immune signalling in the IFNAR or STAT1 knockout mice affects the kinetic expression profiles of lncRNAs during SARS-CoV infection, and the similar changes are also observed in response to both IAV infection and IFN treatment. These data suggest that the differential regulation of lncRNA expression represents a host response as part of the innate immunity and is associated with pathogenic outcomes of IAV infection [18]. These findings are further supported by Josset et al. who performed total RNA-Seq on virus-infected lungs from eight mouse strains. They found that 5329 lncRNAs were differentially expressed after IAV (A/Puerto Rico /8/1934) or SARS-CoV (MA15) infection [19]. Microarray and RNA sequencing assays were also performed in human A549 lung epithelial cells, with or without IAV infection [20,21]. Different expression levels of lncRNAs were reported between the two groups. These lncRNAs regulate cellular metabolic processes, immunity, and autophagy during A/swine/Zhejiang/04 (H3N2) infection [20], and are involved in the α, β, and γ IFN and immune signalling pathways during A/ Puerto Rico/8/1934 infection [21]. Furthermore, Chai et al. showed that 139 lncRNAs were upregulated and 150 lncRNAs were downregulated in both A549 and HEK293T cells after infection, by IAV A/WSN/1933, A/Puerto Rico/8/1934, and A/California/04/2009 [22]. These distinct expression profiles of lncRNAs in cells infected with different strains of IAV suggest an association between the expression of lncRNAs and host susceptibility to different IAV infections [22]. In support of the above observations, deep sequencing of lung RNA in mice that were challenged with a highly pathogenic (A/Chicken/Jiangsu/k0402/2010) or a much less virulent (A/Goose/Jiangsu/k0403/2010) avian influenza virus H5N1 also showed distinct expression of numerous lncRNAs in response to these two viruses, and the lncRNA profiles are correlated with viral pathogenicity in mice [23]. These comprehensive studies have used different approaches to investigate the response of lncRNA expression to IAV infection under various conditions, including infection with different IAV strains, avian influenza virus with different pathogenicity, in different cell lines, or in mice. Changes in lncRNA expression are associated with the host susceptibility to IAV and viral pathogenicity, also determine the outcome of influenza virus infection. Overall, these studies establish a strong association between cellular lncRNA expression and influenza virus infection and suggest their potential utility as a novel diagnostic tool for IAV infection.

## Roles of lncRNAs in influenza virus infection

**Figure 1.**
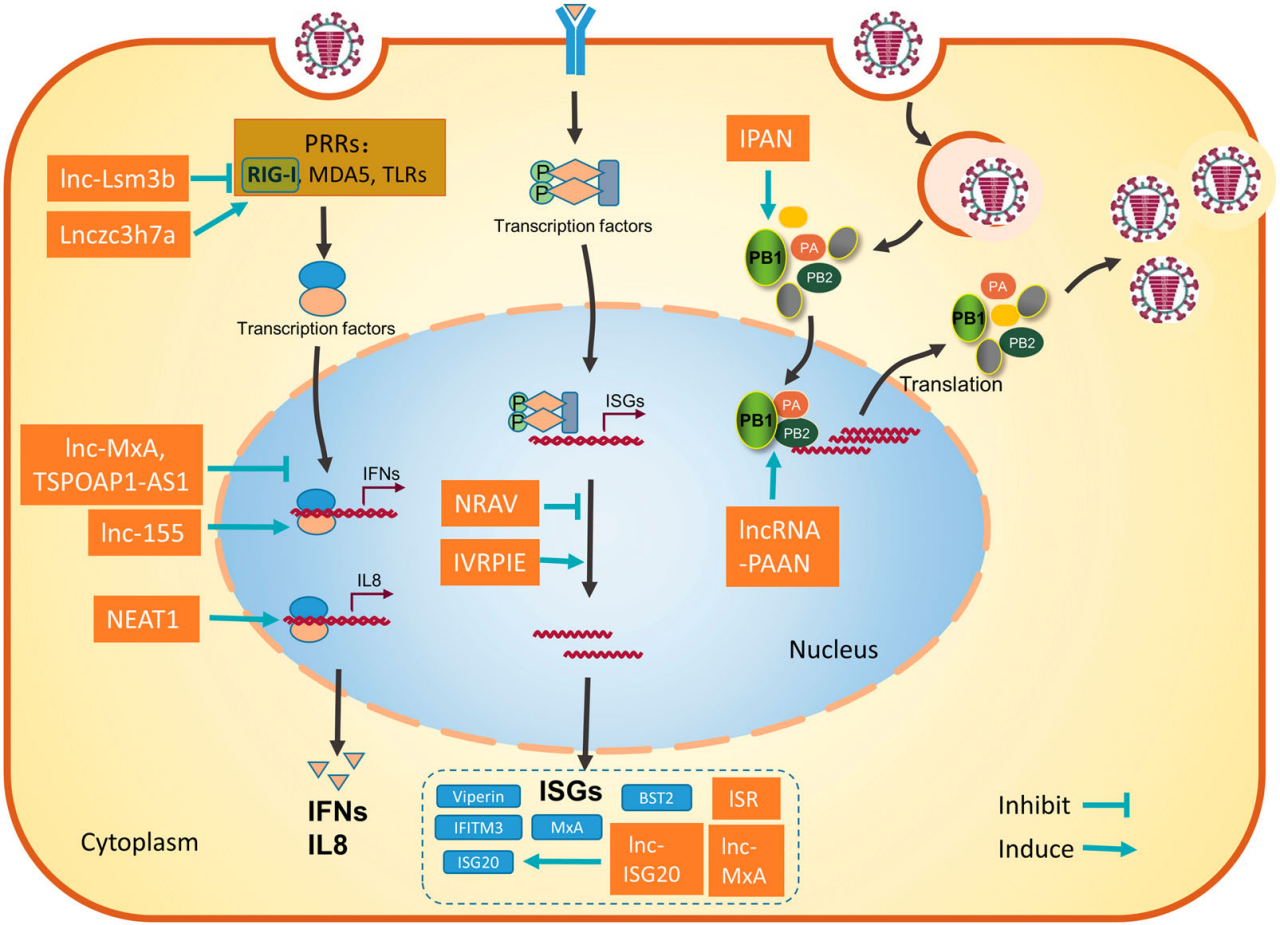
Schematic diagram of the roles of host lncRNAs during influenza virus infection. Most lncRNAs regulate the host immune response against influenza virus at different steps to promote or inhibit the virus infection (left). LncRNAs can also be hijacked by the influenza virus to enhance viral replication (right). These functional lncRNAs are represented as rectangles. Refer to the text for more details.

Following influenza virus infection, the host innate immune system generates the first line of defense and has an important role in clearing the viral infection. In the meantime, the influenza virus has also evolved diverse strategies to evade host innate immunity and/or hijack host factors to establish successful replication [24–26]. Host lncRNAs have been reported to function as either positive or negative regulators of the innate antiviral response and may either inhibit or facilitate viral infection. It is not surprising that lncRNAs are also hijacked by influenza viruses to aid viral replication (Figure 1).

### LncRNAs modulate anti-IAV innate immune response

Host anti-IAV immune response begins with the recognition of conserved viral components called pathogen-associated molecular patterns (PAMPs) by host-pathogen recognition receptors (PRRs), including cytoplasmic retinoic acid-inducible gene I (RIG-I), melanoma differentiation factor 5 (MDA5), and endosomal toll-like receptors (TLRs) [27–29]. This leads to the activation of transcription factors, including IRF3/7 and NF-κB that rapidly trigger the expression of type І and type III interferons (IFNs), which are the major IFNs that are secreted by dendritic cells, macrophages and airway epithelial cells upon influenza virus infections. IFNs interact with their receptors, which in turn activates the JAK-STAT signalling pathway and induces the expression of numerous antiviral proteins encoded by IFN-stimulated genes (ISGs) [30,31]. ISGs, including MxA, IFITMs, and TRIM proteins, block the early stage of viral infection [32–35]. ISGs also inhibit viral mRNA expression and protein translation, including ZAP, OAS-RNase L, PKR, and ISG15 [36–39], and restrict viral release, including viperin and tetherin [40,41]. In contrast, type II interferon is secreted by activated T lymphocytes and NK cells, and mainly modulates the adaptive immune responses against influenza virus infection [42,43]. IFN-γ has been shown to assist CD8+ T cells to differentiate into CTLs and enhance the T-cell proliferation during influenza virus vaccination [24].

#### LncRNAs enhance IAV infection by suppressing the expression of IFN and anti-IAV ISGs

Several studies have shown that many differentially expressed cellular lncRNAs are involved in the host immune response [18,19,21,44]. Some of these lncRNAs actually act as positive regulators of viral infection by inhibiting different steps of host immune response [45].

LncRNAs can regulate the expression of ISGs by multiple mechanisms. Ouyang et al. showed that lncRNA NRAV is dramatically downregulated during IAV infection [46]. NRAV promotes IAV replication likely by suppressing the initial transcription of several key ISGs, including IFITM3 and MxA through affecting the histone modifications H3K4me3 and H3K27me3 of these genes. In support of the positive role of NRAV in influenza virus replication, ectopic expression of human NRAV in mice renders them more susceptible to IAV infection, including faster body weight loss, lower survival, and higher lung viral titre in NRAV transgenic mice compared with wild-type littermates [46]. Another lncRNA TSPOAP1-AS1 is upregulated by IAV infection in a dose- and time-dependent manner [47]. TSPOAP1-AS1 promotes IAV replication by suppressing IAV-induced IFNb1 transcription, interferon-sensitive response element (ISRE) activation, and downstream ISGs expression [47]. lnc-MxA facilitates the replication of IAV by forming an RNA–DNA triplex with the IFN-β promoter, interfering with the enrichment of IRF3 and p65 at the IFN-β promoter and thus negatively regulating the transcription of IFN-β. The activities of lnc-MxA as both an ISG and a negative regulator of the antiviral response suggest the important role of lnc-MxA in maintaining immune homeostasis [48].

Another IFN-inducible lncRNA, lnc-Lsm3b, has also been shown as an active maintenance mechanism of immune homeostasis through directly targeting the viral RNA sensor RIG-I. lnc-Lsm3b contains multivalent structural motifs and a long-stem structure, thus can compete with viral RNAs in binding RIG-I. This interaction of lnc-Lsm3b with RIG-I restricts RIG-I activation and inhibits downstream signalling, as a result, prevents the production of type І IFNs. This function of lnc-Lsm3b was further validated by the study showing higher levels of IFN-β and IL-6 in lnc-Lsm3b-deficient mice which were infected with influenza virus strain A/Puerto Rico/8/1981 through intranasal infection [49]. These data support the concept that host lncRNAs can act as negative regulators of immune response and play important roles in the precise control of the antiviral response. In the context of influenza virus infection, lncRNAs can prevent overactivation of the immune response, thereby avoid host tissue damage which can be caused by potential cytokine storms. Understanding the innate immune modulation activities of these lncRNAs is expected to open new avenues to the design of drugs to treat influenza virus infection and autoimmune inflammatory diseases.

#### LncRNAs inhibit IAV infection by increasing the expression of IFN and anti-IAV ISGs

Host uses lncRNAs to enhance the immune response and inhibit the influenza virus infection. A cytoplasmic lncRNA Lnczc3h7a binds to both TRIM25 and the activated RIG-I at early stages of viral infection. Lnczc3h7a acts as a molecular scaffold to stabilize the TRIM25/RIG-I interaction, enhance TRIM25-mediated K63-linked ubiquitination of RIG-I, and thereby promote RIG-I downstream signalling and the antiviral innate immune response [50]. MIR155HG-derived lncRNA-155 is markedly enhanced by IAV infection and strongly affects IAV replication and virulence. LncRNA-155 has been shown to profoundly inhibit the expression of protein tyrosine phosphatase 1B (PTP1B) during IAV infection and resulting in higher expression of IFN-β and several critical ISGs, thus promoting the innate immune response to viral infection. The immune regulatory function of lncRNA-155 was further demonstrated by a study showing that MIR155HG KO mice are hypersensitive to IAV infection and present more severe lung injury than wildtype mice, because of lower expression of IFNs, critical cytokines, ISGs [51]. A recent analysis of existing transcriptome dataset of patients with IAV infection identifies a novel lncRNA IVPRIE which inhibits IAV replication through promoting the transcription of IFN-β1 and several ISGs including IFIT1, IFIT3, IRF1, ISG15 and Mx1. It is shown that hnPNP U interacts with IVRPIE and is involved in IVRPIE-mediated regulation of histone modifications of IFN-β1 and several ISGs [52].

In addition, lncRNAs can activate the expression of antiviral genes by relocating or competitively binding to gene repressors. For example, the NEAT1 lncRNA is an essential component of paraspeckles which are dynamic subnuclear structures and are dependent on RNA polymerase II transcription [53–55]. Within paraspeckles, NEAT1 binds to splicing factor proline/glutamine-rich (SFPQ/PSF) and is involved in the expression of several innate immune-related genes [56,57]. When NEAT1 is at low levels, SFPQ/PSF acts as a repressor of the IL8 promoter. During IAV infection, NEAT1 expression increases, leading to the relocation of SFPQ/PSF from the IL8 promoter to paraspeckles, and transcriptional activation of the antiviral gene IL8 [57]. Since SFPQ/PSF is also an essential factor for influenza virus mRNA polyadenylation [58], NEAT1 is thus a dual regulator of viral infection through increasing the expression of antiviral genes and facilitating viral gene expression. Another IAV-upregulated lncRNA, lnc-ISG20 shares most of its sequence with ISG20, is a novel ISG and inhibits IAV replication. lnc-ISG20 acts as a competitive endogenous RNA (ceRNA), binds miR-326 to reduce its inhibition of ISG20 translation [22]. In addition, lncRNA ISR is also induced by IAV infection and by IFN-β, thus functions as an ISG to inhibit IAV replication [59].

Taken together, a group of lncRNAs inhibit IAV infection by enhancing the expression of antiviral genes. The underlying molecular mechanisms vary, from directly interacting with signalling molecules, regulating histone modifications of antiviral genes, affecting the expression and relocation of gene repressors, or sequestering microRNA. These lncRNAs thus present promising targets for developing antiviral strategies through strengthening innate immune responses.

### LncRNAs directly participate in IAV replication

The genome of IAV is a set of eight negative single-strand RNA segments, each of which is associated with the nucleoprotein (NP) and three subunits (PB1, PB2 and PA) of the RNA-dependent RNA polymerase (RdRp) complex. Following virus endocytosis and fusion of cellular and viral membranes, the viral ribonucleoproteins (vRNPs) are released into the cytoplasm and further transported into the nucleus, where viral RNA transcription and replication take place. The incoming vRNPs first produce the primary mRNAs, and then the essential proteins for viral RNP replication (NP, PB1, PB2, and PA) are synthesized. The newly assembled vRNPs are then transported out of the nucleus, assemble at and bud from the host membrane [60–62]. IAV has evolved to hijack host lncRNAs at different stages of the viral life cycle so as to complete a successful infection.

Results of a loss-of-function screen identified a novel group of lncRNAs that modulate IAV replication [63], among which two IFN-independent host lncRNAs (IPAN and PAAN) were further characterized, and both were shown to be significantly induced by IAV infection. LncRNA IPAN was shown to associate with viral PB1 protein, prevent PB1 degradation, thus promoting IAV transcription and replication [63]. Instead, the other lncRNA, PAAN, associates with viral PA protein and promotes the assembly of viral RNA polymerase, thus warranting efficient vRNA synthesis [64]. These findings demonstrate that specific lncRNAs are exploited by IAV as important host factors to facilitate viral replication at key steps of the viral infection.

### LncRNA promotes IAV replication by modulating cellular metabolism

Replication of influenza virus requires energy for the synthesis of viral proteins, viral RNA, or specific membrane lipids [65]. There are studies showing that IAV infection increases glucose uptake, glycolysis, and lactic acid production [66,67]. Cao et al. showed that IFN-independent lncRNA-ACOD1 is induced by multiple viruses, including IAV, and promotes IAV replication in A549 cells. LncRNA-ACOD1 directly binds GOT2 at a site close to the substrate niche and simulates GOT2 catalytic activity and production of its metabolites, thus facilitating viral replication [68]. This example reveals one strategy by which viruses indirectly utilize lncRNAs to modulate host metabolic pathways and ensure viral survival.

## Some lncRNAs modulate IAV infection by uncharacterized mechanisms

In addition to the above relatively well-studied lncRNAs, some lncRNAs have also been shown under the influence of influenza virus infection, but the exact roles of these lncRNAs in IAV infection remain to be elucidated. VIN, a virus-inducible lncRNA, has been shown to be upregulated by IAV, but not by influenza B virus, treatment with RNA mimics, or IFN-β. The depletion of VIN results in reduced IAV production and decreased viral protein synthesis, suggesting that VIN is important for productive IAV infection [69]. Similarly, another lncRNA, PSMB8-AS1, is also induced by IAV infection and IFN-β1, and depletion of PSMB8-AS1 reduced the expression of IAV genes and the release of progeny IAV virions [21]. However, it is largely unclear how VIN and PSMB8-AS1 modulate IAV infection. In any case, their nuclear localization suggests their roles in the transcription and/or replication of the IAV RNA genome.

In addition, more lncRNAs, such as lncBST2/BISPR [70–72], lncISG15 [70], and EGOT [73], are differentially expressed upon infection with different viruses including IAV. These lncRNAs regulate host antiviral pathways or suppress the replication of viruses other than IAV. The detailed molecular mechanisms behind the actions of these lncRNAs await further investigation.

## Conclusions and perspectives

Over the past 10 years, thousands of lncRNAs have been shown to be differentially expressed in either human cells or mice in response to IAV infection. It is posited that lncRNA expression profiles are likely associated with IAV virulence and host susceptibility to different IAV strains. Several lncRNAs lncISG15, lncBST2, ISR2 and ISR8 can be significantly induced by the infection of NS1 null IAV (PR8ΔNS1) which is unable to block the IFN response [70,74]. This suggests the importance of the NS1 protein in the regulation of lncRNA expression. Expression of lncRNAs can have also been changed in response to the expression of viral protein from the Hepatitis B virus and human immunodeficiency virus [75,76]. Despite these progress, the interaction of IAV proteins and lncRNAs is still completely understood. With many protein factors identified to interact with IAV proteins, characterization of lncRNA expression profiles in IAV infection and the interaction of the lncRNAs with specific viral proteins will advance our understanding of the spatial and temporal regulation of lncRNAs and discover speciﬁc potential drug targets.

As mentioned above, lncRNAs affect IAV infection by varied mechanism. They either positively or negatively regulate host immune responses to IAV infection, they can be directly hijacked by IAV to enhance viral replication, or they can modulate viral infection though altering cellular metabolism. These diverse mechanisms of action by these lncRNAs in the context of IAV infection are summarized in Table 1, which highlight the active and important roles of lncRNAs in IAV–host interactions. However, research on the specific lncRNA functions and mechanisms during IAV infection is still at its early stage, and more work needs to be done. Detailed functional studies are required to elucidate the landscape of host lncRNAs-IAV interplay and to understand the lncRNA-mediated regulatory network in IAV infection.

**Table 1.** Roles of lncRNAs in modulating IAV infection.

LncRNAs	Influenza strains	Screening methods	Functions	Mechanisms	Subcellular localization	Neighbors	Reference
NRAV	A/WSN/1933 (H1N1)	Genome-wide lncRNA microarray	Positive	Negatively modulates antiviral responses by suppressing the initial transcription of several key ISGs, including IFIT2, IFIT3, OASL, IFITM3 and MxA	Nucleus	DYNLL1	[46]
TSPOAP1-AS1	A/Puerto Rico/8/1934 (H1N1)	Unknown	Positive	Represses IAV-triggered type I IFN signaling by negatively regulating induction of several anti-IAV ISGs, including IFIT1, IFITM1, OASL and ISG20	Nucleocytoplasmic lncRNA, and IAV infection promoted its nuclear localization	Unknown	[47]
Lnc-MxA	A/WSN/1933 (H1N1)	RNA deep sequencing	Positive	Is an ISG and inhibits the activation of IFN-β transcription by forming an RNA-DNA triplex at its promoter	Cytoplasm and translocates to nucleus in IAV-infected cells	MxA	[48]
Lnc-Lsm3b	A/Puerto Rico/8/1934 (H1N1)	UV-RIP-seq with Flag-RIG-I	Positive	Competes with viral RNAs in the binding of RIG-I monomers to restrict RIG-I activation and prevents overproduction of type І IFNs	Cytoplasm	LSM3	[49]
Lnczc3h7a	A/Puerto Rico/8/1981 (H1N1)	UV-RIP-seq with Flag-TRIM25	Negative	serves as a molecular scaffold for stabilization of the RIG-I–TRIM25 interaction, facilitating K60-linked ubiquitination of RIG-I and downstream signaling	Cytoplasm	ZC3H7A	[50]
LncRNA-155	A/WSN/1933, A/Puerto Rico/8/1934 (H1N1)	Genome-wide lncRNA microarray	Negative	Promotes innate immune response by suppression of PTP1B to upregulate IFN-β and several ISGs expression	Nucleus	MIR155HG	[51]
IVPRIE	A/Beijing/501/2009 (H1N1)	Analysis of existing dataset (GSE108807) of RNA sequencing	Negative	Promotes host antiviral immune response through positively regulating the IFN-β and ISGs expression by affecting histone modification of these genes	Nucleus	TANK	[52]
NEAT1	A/WSN/1933 (H1N1)	Unknown	Negative	Enhances transcriptional activation of IL8 through relocating SFPQ from IL8 promoter to the paraspeckles	Nucleus	FRMD8	[57,77]
Lnc-ISG20	A/WSN/1933, A/Puerto Rico/8/1934, and A/California/04/2009 (H1N1)	RNA deep sequencing	Negative	Is an ISG and reduces the miR-326 mediated inhibition of ISG20 expression by binding to miR-326	Cytoplasm	ISG20	[22]
ISR	A/WSN/1933 (H1N1)	LncRNA microarrays	Negative	Participates in host antiviral defense as an ISG	Unknown	BAHCC1	[59]
IPAN	A/WSN/1933 (H1N1)	An esiRNA- mediated loss-of-function screening	Positive	Promotes IAV transcription and replication by associating with viral PB1 to enhance its stability	Cytoplasm and IAV infection promoted its nuclear localization	PKN2	[63]
LncRNA-PAAN	A/WSN/1933 (H1N1)	An esiRNA- mediated loss-of-function screening	Positive	Promotes the assembly of RdRp complex and thereby enhances viral RNA polymerase activity	Cytoplasm and IAV infection promoted its nuclear localization	TCAIM,ZNF445	[64]
LncRNA-ACOD1	A/Puerto Rico/8/1934 (H1N1)	RNAi-mediated functional screening	Positive	Stimulates GOT2 catalytic activity and production of its metabolites	Cytoplasm	ACOD1	[68,69]
VIN	A/WSN/1933 (H1N1)	NCode™ and Sureprint™ G3 microarrays	Positive	Unknown	Nucleus	ACTR3	[69]

## Abbreviations

Abbreviations: CTL, cytotoxic T lymphocyte; EGOT, eosinophil granule ontogeny transcript; GOT2, glutamic-oxaloacetic transaminase; hnPNP U, heterogeneous nuclear ribonuclear protein U; IAV, influenza A virus; IFITM, interferon-induced transmembrane protein; IFN, interferon; IFNAR, interferon-α/β receptor; IPAN, influenza virus PB1-associated noncoding RNA; IRF, interferon regulatory factor; ISG, IFN-stimulated gene; ISR, interferon-stimulated lncRNA; ISRE, interferon-sensitive response element; IVPRIE, inhibiting IAV replication by promoting IFN and ISGs expression; JAK-STAT, Janus kinase-signal transducer and activator of transcription; lncRNA, long non-coding RNA; lncBST2/BISPR, BST2 IFN-stimulated positive regulator; MDA5, melanoma differentiation-associated gene 5; MxA, Myxovirus resistance protein 1; NEAT1, nuclear enriched abundant transcript 1; NRAV, negative regulator of anti-viral; OAS-RNase L, 2′-5′-oligoadenylate synthetase-ribonuclease L; PAMP, pathogen-associated molecular pattern; PB1, polymerase basic protein 1; PA, polymerase acidic protein; PAAN, PA-associated noncoding RNA; PKR protein kinase R; PRR, pattern recognition receptor; PTP1B, protein tyrosine phosphatase 1B; RIG-I, retinoic acid-inducible gene 1; SFPQ/PSF, splicing factor proline/glutamine-rich; TLR, toll-like receptor; TRIM, tripartite motif-containing; VIN, virus-inducible lincRNA; ZAP, zinc-finger antiviral protein
